# Fast, accurate, and cost-effective poultry sex genotyping using real-time polymerase chain reaction

**DOI:** 10.3389/fvets.2023.1196755

**Published:** 2023-11-03

**Authors:** Ciro D. Cordeiro, Nesim Gonceer, Steve Dorus, James E. Crill, Vardit Moshayoff, Amit Lachman, Asaf Moran, Dan Vilenchik, Shlomit Fedida-Metula

**Affiliations:** ^1^N. R. Soos Technology, Kidron, Israel; ^2^Department of Biology, Center for Reproductive Evolution, Syracuse University, Syracuse, NY, United States; ^3^Forensic and National Security Sciences Institute, Syracuse University, Syracuse, NY, United States; ^4^OVO Technology, Kidron, Israel; ^5^School of Electrical and Computer Engineering, Ben Gurion University of the Negev, Beer-Sheva, Israel

**Keywords:** real-time PCR (qPCR), sex genotyping, gonad, poultry, breed

## Abstract

According to The Organization for Economic Co-operation and Development (OECD), demand for poultry meat and eggs consumption is growing consistently since poultry meat and eggs are readily available and cheap source for nutritional protein. As such, there is pressing demand from industry for improved protocols to determine chicken sex, especially in layer industry since only females can lay eggs. Extensive efforts are being dedicated to avoiding male chicks culling by developing *in-ovo* sexing detection methods. Any established *in-ovo* detection method will need to be validated by embryo genotyping. Therefore, there is a growing demand for fast, inexpensive, and precise method for proper discrimination between males and females in the poultry science community. Our aim with this study was to develop an accurate, high-throughput protocol for sex determination using small volumes of blood. We designed primers targeting the Hint-W gene within the W chromosome clearly distinguishing between males and females. In the interest of establishing an efficient protocol without the need for gel electrophoresis, crude DNA extraction without further purification was coupled with qPCR. We validated the accuracy of our method using established protocols and gonad phenotyping and tested our protocol with four different chicken breeds, day-nine embryos, day-old chicks and adult chicken. In summary, we developed a fast, cost-effective, and accurate method for the genotyping of sex chromosomes in chicken.

## Introduction

The poultry industry (meat and eggs) is an important and essential source of healthy nutritious proteins. This growing demand for healthy and inexpensive meat is due to a significant increase in world population (1). The increase of eggs (layers industry) and poultry meat consumption (broiler industry) is happening in the developing world, in part, because of higher incomes and recent awareness for better diets consisting higher proteins level (2), the largest eggs *per capita* consumers were Japan, United States, Paraguay, and China (3) and greater demand is expected in the future.

Practices in the layers industry are remarkably insufficient. Unlike the broiler industry, which produces meat products from both sexes, the layers industry focuses on female chicks only for their ability to lay eggs. The male chicks are culled immediately post hatching since they do not produce eggs and their meat production is low compared to broiler breeds. In recent years, there was a growing awareness to day-old male chick culling for economic and animal welfare reasons. During 2022, Germany, France and Spain banned culling male chicks in the layers industry, and other European countries are expected to follow. To address these concerns, technologies are being developed for sex detection in early stage of embryonic development prior to hatching while maintain high egg hatchability after sexing. Some methods are based on differences in embryonic hormonal profiles (4–7), phenotypic characteristics such as extra-embryonic blood vessels (8, 9), embryonic sex detection by magnetic resonance (10, 11) or even by audio technology (12). Any developed detection method must be validated by genotyping of the embryos to evaluate the methodology’s accuracy. One challenge for genotyping is how to process massive quantities of samples efficiently. Previously published methods for sexing by genotyping are available but most rely on Polymerase Chain Reaction (PCR) followed by gel electrophoresis to distinguish between males that have two Z sexual chromosomes (ZZ) and females that have one Z and one W sexual chromosome (ZW). These PCR based approaches require both pre- and post-processing steps which are time-consuming and more expensive. For example, a common PCR method is based on diagnostic differences in intron length of the Chromo-Helicase-DNA-binding 1 (CHD-1) gene (13–16) located on Z and W chromosomes. However, this method requires pre- and post-PCR processes, i.e., genomic DNA (gDNA) purification, measurement of concentration, electrophoresis, and image analysis. Therefore, longer time is required and limited by the number of samples processed at once. Another published Quantitative-PCR (qPCR) based method amplifies Double and Mab-3 related transcription factor-1 (DMRT1) sequence (17) which resides on the Z sex chromosome and Xho-I repeats on W chromosome for clear discrimination between males and females. This method requires purification and clear concentration measurements of DNA samples before performing the assay. Commercial kits are also available (i.e., Spin-W TaqMan) but are much more expensive and require purification of gDNA.

**Figure 1:**
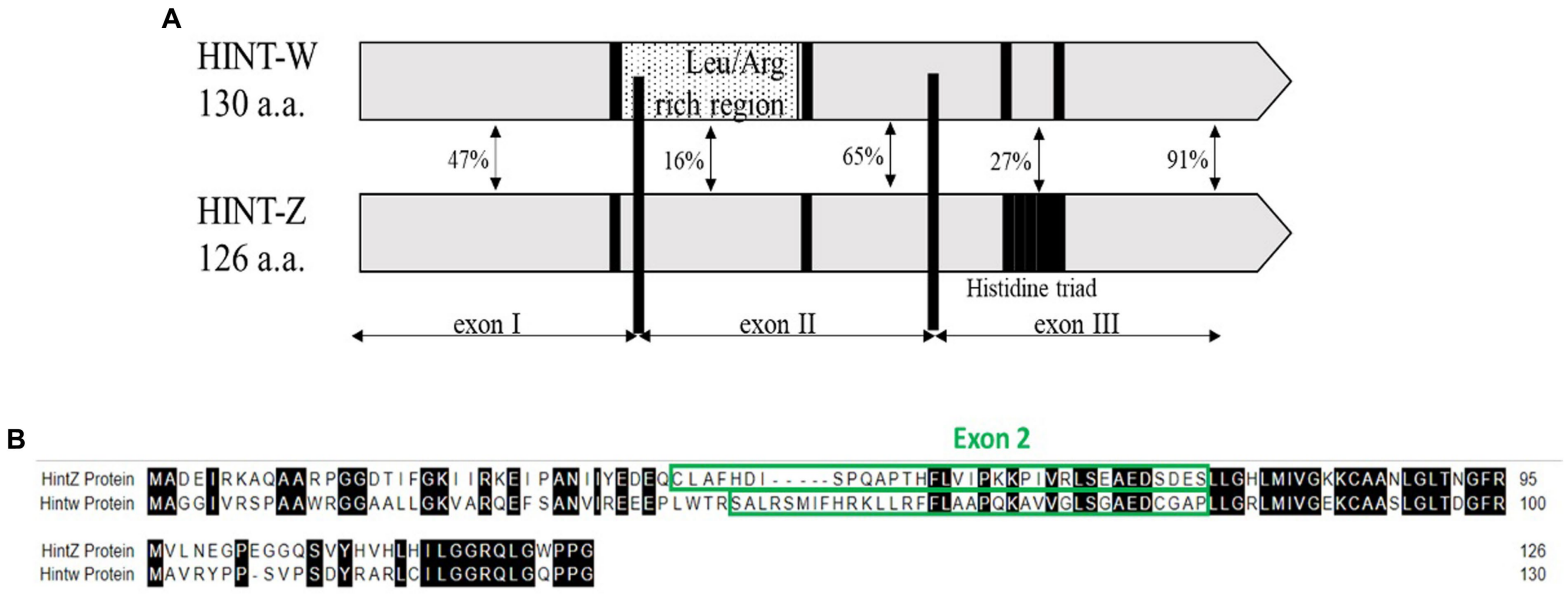
**(A)** Schematic view depicting homology regions of chicken Hint-W and Hint-Z proteins. Histidine triad motif resides on Hint-Z only (black square) while Leucine-Arginine rich region resides on Hint-W only and was the basis for primers design (dotted box) [Modified from Hori et al. (18)]. **(B)** Sequence alignment of Hint-W and Hint-Z genomic sequences.

We developed a method based on the genetic differences between males (Z) and females (W) sex chromosomes which are detected by amplification of a unique sequence that resides in the W-linked HINT-W gene that has low homology on HINT-Z gene on Z chromosome (Figure 1A). HINT-W is unique to female birds for being W-linked gene and widely expressed in female chicken embryo, mainly in gonads and central nervous system (19) which suggests a key role in sex-differentiation. Briefly, the gene name stands for the Histidine triad Nucleotide binding protein W-linked. HINT genes encode the family of nucleotide hydrolase enzymes which carry three histidine motifs (His-x-His-x-His-xx) (Figure 1A). These enzymes are capable of hydrolyzing molecules of Adenosine-5 mono-phosphoramidate (AMP-NH_2_) or AMP-Lysine molecule (20, 21). Unlike HINT-Z protein, HINT-W protein lacks the three Histidine motif which makes it unable to hydrolyze AMP molecules (18, 22), leaving the function of HINT gene to the full version encoded by HINT-Z. In addition to the lack of Histidine triad motif, HINT-W characterized by a Leucine/Arginine rich region which holds only 16% homology to the HINT-Z protein sequence (18) (Figure 1B). Besides those two regions, most coding regions of HINT-Z and HINT-W sequences are well conserved.

Overall, most of the already available methods for sex identification do not meet all requirements of a diagnostic assays in terms of sensitivity, accuracy, rapidity and low-cost. In this study, we present a fast and cost-effective method for genotyping sex chromosomes accurately using Real Time PCR (qPCR). Additionally, we optimized a blood lysis into a one-step method that precludes the need for extra steps of DNA purification. This sex determination method was compared to a commercial TaqMan based qPCR for different poultry breeds and was found to be accurate, fast, and cost-effective. Our assay was extensively evaluated using hundreds of embryos, day-old chicks and adult chickens and validated by crossing genotyping results with the phenotype of gonads. The protocol presented here is ideal for large number of samples and high-throughput applications.

## Materials and methods

### Incubation and hatching

Fertilized eggs for layers and broilers of different breeds were purchased from commercial suppliers. Breeds used for the experiments are DeKalb white, Hy-Line and Bovan Brown (Hendrix Genetics, Boxmeer, Netherland) and Ross308 (Aviagen, Alabama, United States). Eggs were incubated in a designated incubator (EMKA Incubators, Kuurne, Belgium) equipped with an automatic tilt system and set for 37°C and 55% humidity and 45^o^ tilt every 50 min. On day 9 of incubation, fertility check was performed by illumination of each egg with a flashlight. Infertile eggs were discarded while 312 eggs containing developing embryos were removed from incubator and stored at 4°C until they were processed. The rest of the fertile eggs (1789 total in several different experiments) continued incubation and on day 18 were transferred to hatching baskets for additional 3 incubation days till hatching (21 days). Upon hatching, each chick was leg-tagged with a small, numbered plastic tag.

### Sample collection and lysis

Experimental procedures with nine-day embryos and chicken were performed according to the guidelines from the American Society of Animal Science (23). Embryos were decapitated before dissection and one day-old chicks’ euthanasia was performed by cervical dislocation. Blood collection of adult chicken and one-day-old chicks was performed using 4 mm animal Lancet blade (Medipoint Inc., Mineola, New-York, United States). Three microliters of blood sample were collected from small vein in the upper foot of each chick with minimal pain or damage to the skin. For embryo blood collection, eggs were incubated for 9 days and then cracked open, 3 μL of blood was taken from blood vessels of each embryo (this procedure was fatal). Each blood sample was transferred to the bottom of a designated V-shape well of 96 well polystyrene plate (Greiner Bio-one GmbH, Kremsmunster, Austria), sealed with temperature resistant sealing plastic (SPL life sciences, Gyeonggi-do, Korea) and stored at –20°C condition. Red blood cells of chicks are nucleated, therefore there is no need for special white blood cells separation. For lysis of blood cells, 90 μL of 0.05 M NaOH (Romical, Beer-Sheva, Israel) was added to each well, plate was sealed with temperature resistant plastic cover and left in room temperature for 5 min for proper lysis. Plate was incubated at 95°C for 10 min in a C1000 Thermo-cycler (Bio-Rad Laboratories, Hercules, California, United States) to release the DNA from nuclei. After cooling of the sample plate, 10 μL of 1 M Tris pH 7.5 (Hy Laboratories, Rehovot, Israel) was added for neutralization of high pH levels of NaOH.

### Real-time PCR (qPCR) analysis – Hint-W amplification

For qPCR reaction, 3.6 μL of nuclease free water-PCR grade (Hy Laboratories, Rehovot, Israel) was mixed with 5 μL iTaq^™^ Universal SYBR Green qPCR Master Mix (Bio-Rad Laboratories, California, United States) and 0.2 μL (100 nM) of each of the primer for Hint-W genomic sequence (Integrated DNA Technologies, Leuven, Belgium) and 1 μL of lysate (1% of total lysate). Amplicons obtained are 90 base-pairs (bp) long, matching amplification through qPCR. For better assessment of reliability of the method in high throughput sampling and analysis, crude genomic DNA was not purified nor quantified.

We used qPCR Step one plus (Thermo-Fisher Scientific, Waltham, Massachussetts, United States) and conditions were as follows: pre-denaturation by 95°C for 2 min (holding stage), 30 cycles composed of denaturation 95°C for 5 s followed by annealing at 61°C for 30 s (cycling stage) annealing temperature was matching for primer sequences of Hint-W (HTF1) – forward: 5′ – ATA TTT CAC CGC AAG CTC CTA C – 3′ and Hint-W (HTR1) – reverse: 5′ – AGG TGC GCC ACA ATC TTC – 3′. For melting curve analysis, we started with 95°C for 15 s followed by 61°C for 1 min and 95°C for an additional 15 s. For each cycle in the melting curve, the annealing temperature increased by 0.3°C.

### Real-time PCR (qPCR) analysis – validation of results using Spin-W amplification

Commercial kit based on TaqMan primers for amplification of Spin-W were used for validation of the method. In summary, 100 ng of gDNA purified from crude whole blood lysate was mixed with 5 μL of TaqMan^®^ Fast Advanced Master Mix (2X) (Thermo-Fisher Scientific, Waltham, Massachussetts, United States) and 0.5 μL of each of the Spin-W (SPIN1W: Gg03813967-s1) primers (20X) (Thermo-Fisher Scientific, Waltham, Massachussetts, United States) designed for Spin-W genomic sequence with FAM reporter dye. 10 μL final volume was adjusted using Ultrapure Dnase/Rnase Free distilled water (Thermo-Fisher Scientific, Waltham, Massachussetts, United States). Samples were subjected to the qPCR using amplification protocol according to manufacture recommendations. Since TaqMan^®^ primers are used, no melting curve is provided.

### Validation of results using PCR for CHD1-Z and CHD1-W

The second molecular technique to validate our results was PCR assay using primers for CHD1 gene called InSex-F and InSex-R (13). We used whole crude blood lysates as templates for the CHD1 PCRs (although, purified gDNA is recommended). The PCR assay was performed as recommended by Taq DNA Polymerase manufacturer (Bio-Lab, Jerusalem, Israel). Briefly, we used 0.2 mM dNTPs, 1X Standard Taq Buffer, 0.2 μM InSex primers, 1.25 UI of Taq DNA Polymerase and 2 μL of total crude blood lysate (2%). PCR conditions were as follows: pre-denaturation by 95°C for 2 min (holding stage), 35 cycles composed of denaturation 95°C for 20 s followed by annealing 60°C for 20 s and extension 68°C for 45 s. Final extension was performed at 68°C for 5 min. PCR products were separated by running on 1.5% agarose gel (Lonza Group, Rockland, Mine, United States) and stained with SYBR Safe DNA Gel Stain (Thermo-Fisher Scientific, Waltham, Massachussetts, United States).

### Validation of PCR results based on gonad status through dissection

**Figure 2:**
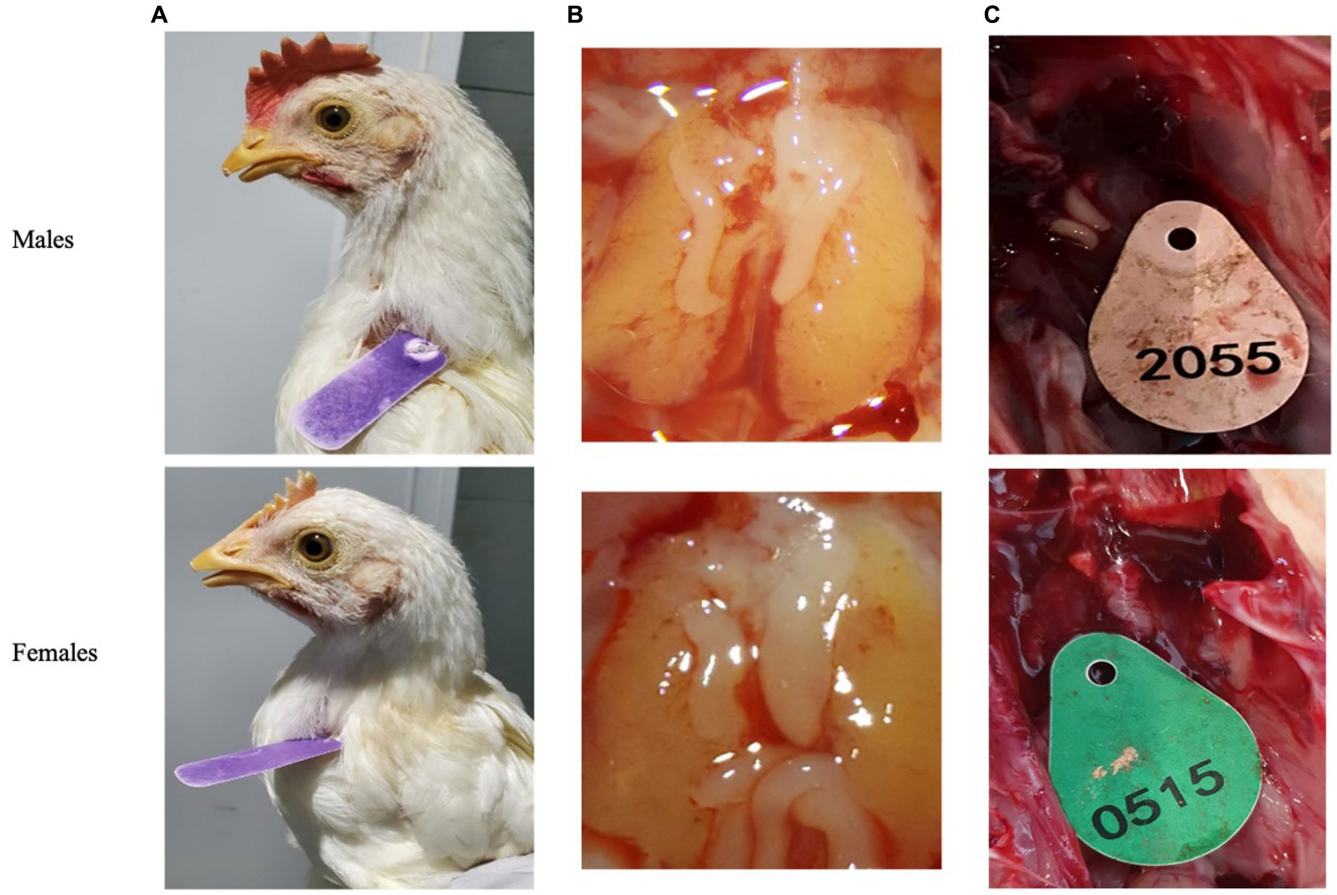
Secondary sexual characteristics were used to validate qPCR genotyping. **(A)** Adult chickens. **(B)** Embryos’ gonads (testicles or ovaries) after 9 days of incubation. **(C)** One day old chicken’s gonads.

Post blood sampling, embryos were dissected using Binocular Zeiss Stereo Stemi DV4 microscope (Carl Zeiss company, Stuttgart, Germany). Day-old chicks were euthanized by cervical dislocation and then dissected for revealing the gonads to validate sex genotyping by various methods of PCR. Clear differences in gonads morphology can be observed on day-9 of embryonic development. Morphology of female gonads (Ovaries) is quite clear when the right ovary undergo atrophy while the left ovary is normally developed and is bigger than right ovary. Alternatively, Morphology of male gonads is a banana-like cylinder shape of symmetrical set of two identical testes (24) (Figures 2B,C).

## Results and discussion

### Primers and qPCR design

The first step for correct genotyping was to find a specific sequence for W chromosome amplification. The qPCR primers design was focused on the Leucine/Arginine region which starts at the end of Exon I and continues to Exon II of HINT-W gene. Because of the low homology between HINT-W and HINT-Z, amplification was expected only for the W-linked gene copy (Figure 1B). For genomic amplification of this region, we focused on the sequence residing solely on Exon II since polymorphisms are less frequent in exonic regions and would be more likely to amplify readable sequences (25). We chose a site with good primer characteristics that generated amplicon size of 90 bp required for qPCR. Primers were named HTF1 (Hint-W Forward) and HTR1 (Hint-W Reverse).

### Genomic DNA purification is not required for sex genotyping

**Figure 3:**
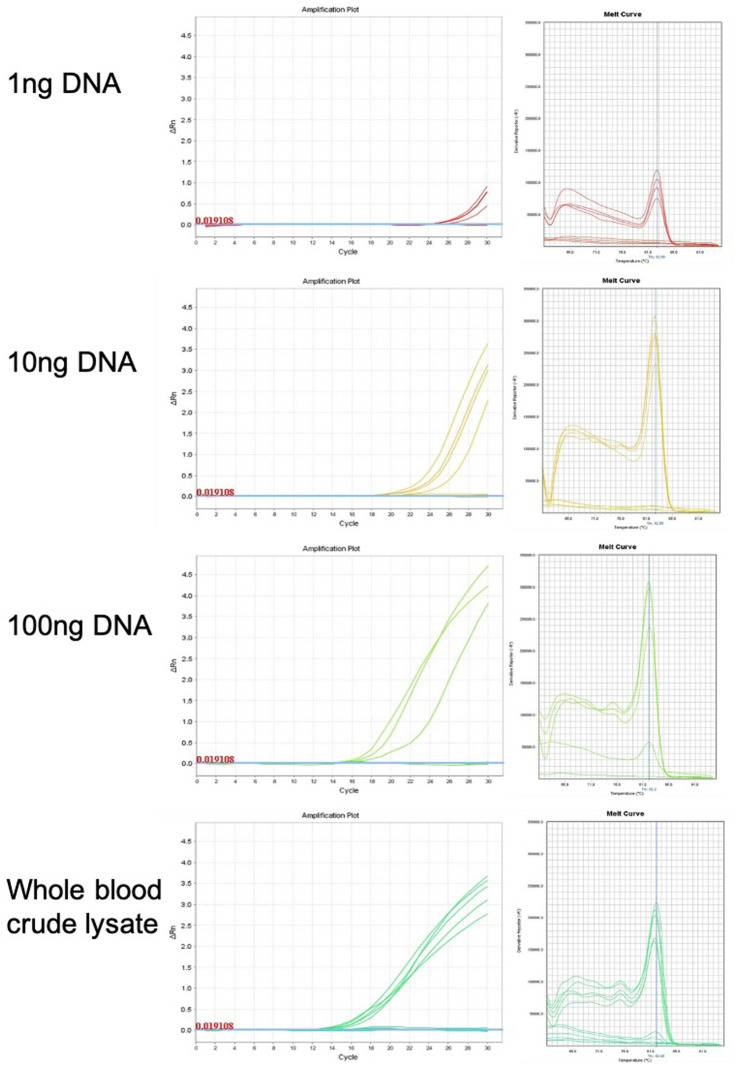
Genomic DNA purification is not required for genotyping PCR reaction. Amplification plots and melt curves of 10 qPCR reactions for each concentration using HWF and HWR primers. Each line corresponds to one sample from male or female chick. Templates used were purified gDNA at indicated concentrations or whole blood crude lysate. We observed equivalent results using crude lysate or purified DNA.

**Figure 4:**
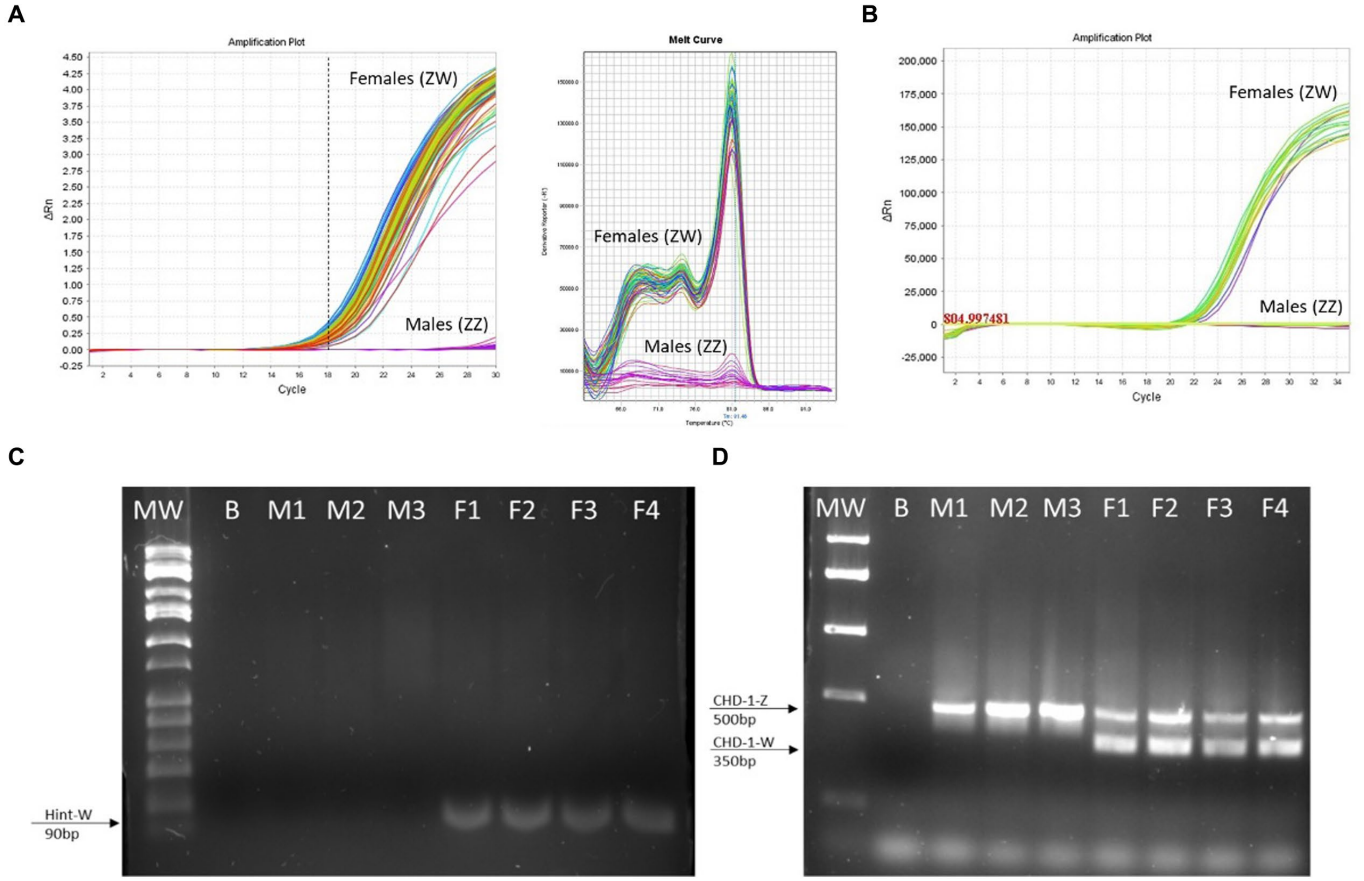
The Hint-W genotyping method is reliable. **(A)** Hint-W amplification plot with melting curve analysis. Each line corresponds to one sample. Observe the clear discrimination between amplification of females and males while melting curve analysis provide clear curve for a single-specific product. **(B)** commercial Kit using TaqMan primers designed to amplify Spin-W gene using purified gDNA for discrimination between females and males. Amplification exists within females while males present flat line without any amplification. The results of A and B are representative of 100 samples tested. No melting curve provided using TaqMan primers. **(C)** Gel electrophoresis of qPCR for amplification of Hint-W gene using HWF1 and HWR1 primers. **(D)** PCR for amplification of CHD-1 gene was performed as previously published by Febriyanti et al. (13). The results in C and D are representative of 50 samples tested. B, blank control without any DNA; M, male samples; F, female samples.

qPCR was used for chicken sexing/genotyping as it does not require post PCR analysis based on electrophoresis and DNA-size migration. We tested the new Hint-W primers HTF1 and HTR1 in PCR reactions with chicken purified gDNA at different concentrations (Figure 3). In our pursuit for a faster processing protocol, we tested a simple blood lysate method using only sodium hydroxide at 95°C. The basic pH and temperature destroy cellular membranes releasing the DNA into solution, which was then neutralized with 1 M TRIS buffer (pH 7.5). The qPCR results obtained with whole cell crude lysate were similar to results obtained with purified DNA (Figure 3) and all female samples were amplified. In addition, the lysate did not inhibit amplification and the primers amplified one single band as evidenced by the melt curve (Figure 3) and confirmed by gel electrophoresis (Figure 4C). All results obtained from male or female chickens were confirmed by inspecting their secondary sexual characteristics, i.e., development of comb, wattle and spur.

### Robust qPCR genotyping for large scale use

Next, we collected 3 μL or 10 μL of crude blood sample from one-day-old chicks to estimate the minimal required volume to avoid damage to the chick. Our results indicated that the Hint-W amplification by qPCR assay allows a clear differentiation between males and females chicks in low and high crude extracts DNA concentration while no PCR inhibition in high concentration was observed (Supplementary Figure S1).

We then tested 100 adult chickens with our new Hint-W detection protocol. We also confirmed the genotyping results by inspecting secondary sexual characteristics and gonads (Figure 2A). The phenotypes observed were in concordance with all results obtained using different methods of PCR. As shown in Figure 4A, samples from females amplified well while samples from males did not. In addition, analysis of the melting curve is important to determine specificity of the reaction. A single peak melting curve in 81°C confirmed that the primers were recognizing only one sequence region within the gDNA, and no additional product was obtained besides the targeted region of the Hint-W (Figure 4C). We then tested the same samples for validation using published PCR methods. The same crude lysates from 100 chicks were used for gDNA purification and then amplified using Spin-W TaqMan commercial primers and qPCR (Figure 4B), which confirmed our previous results. All samples tested in this experiment using Spin-W or Hint-W protocols presented the same genotyping results. Then we performed gel electrophoresis of the Hint-W PCR samples and our results confirmed the qPCR analysis (Figure 4C). Finally, we used the CHD-1 detection technique (13) with InSex-(F) and InSex-(R) primers to validate results from 50 samples (Figure 4D). The gel bands confirmed our qPCR results: Females present double bands (500 bp and 350 bp) originated from different amplicon sizes in Z or W Chromosomes and males present a single band (500 bp). As expected, all samples tested in this experiment using CHD-1 or Hint-W protocols presented the same genotyping results. The summarized protocol for our Hint-W method is provided in Supplementary File 1.

### Validation of qPCR results

To test the accuracy and validate of our method we performed an experiment with 312 embryos incubated for 9 days. Male and female chicken embryos have dimorphic gonads (testicles or ovaries) that can easily be distinguished after 9 days of embryonic development (Figure 2B). We collected blood, dissected the embryos and performed Hint-W qPCR for all embryos. We observed that only 6 (error of 1.92%) were incorrectly classified as male or female while 306 embryos (98%) presented matching gonads to the genotyping results by Hint-W qPCR (Supplementary Table S1). The accuracy for this test was 98%. Interestingly, 5 of the 6 misdiagnosed samples had the Ct score “undetermined” (i.e., below the detection limit). In total, 15 of the samples had Ct score undetermined, 10 were correctly diagnosed as males and 5 were wrongly classified as females. Therefore, we recommend repeating those samples with “undetermined” Ct scores to confirm the results.

### Analysis of Ct scores in qPCR results

**Figure 5:**
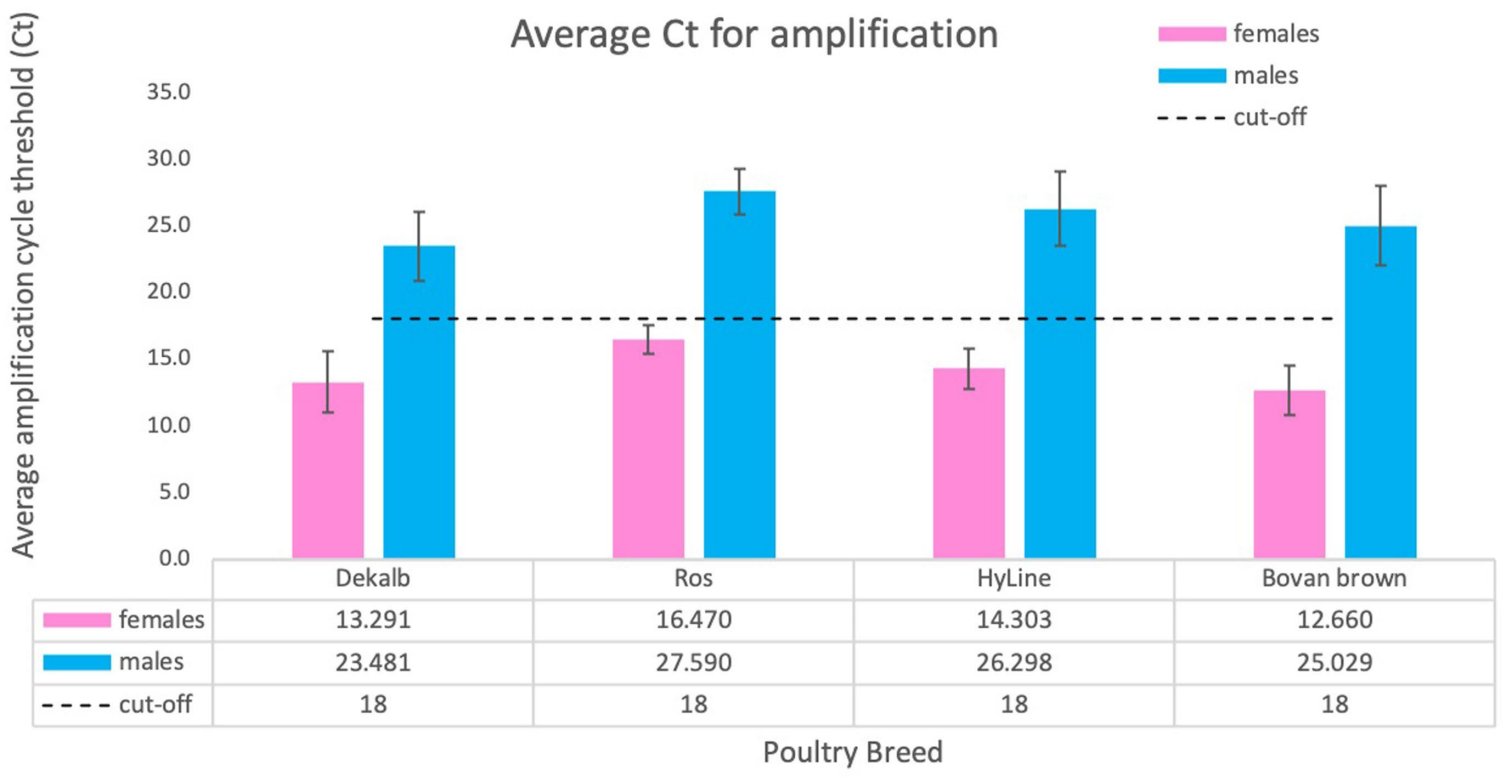
Average Ct score obtained for different chicken breeds. We tested a total of 2,101 animals of four breeds: DeKalb White (*n* = 834), Ross 308 (*n* = 255), Bovan Brown (*n* = 92), HyLine (*n* = 920). Values are average ± SD for one experiment using each breed. The results were analyzed for cycle threshold cut off and general Ct value for females (Ct ≤18) and for males (usually no amplification or Ct ≥ 23).

The determination of cycle threshold cut-off for discrimination between females and males was completed using a total of 2,101 samples tested, including adult chicken, nine-day-old embryos and one-day-old chicks. We used samples obtained from four domestic breeds: DeKalb White (*n* = 834), Ross 308 (*n* = 255), Bovans Brown (*n* = 92), HyLine (*n* = 920). Considering the differences and variability of total amount of gDNA obtained by crude lysate, we conclude that the cut-off value of cycle threshold (Ct) for clear discrimination between females and males is 18 (Figure 5). This number was established by averaging Ct values of all birds from different breeds. Our analysis determined that amplification of females usually takes place before Ct ≤ 18 while amplification, if any, of males will occur much later Ct ≥ 23 (Figures 4A, 5).

### Comparison with other methods

Finally, we compared our Hint-W qPCR based genotyping method with other methods published or commercially available (Table 1). We found that our protocol has several advantages over most PCR-based methods, including fewer steps and easy scalability with a high specificity and sensitivity. For example, our method requires only three steps (blood collection, blood lysis and qPCR), which makes it more efficient and easier to perform, especially for large-scale genotyping experiments. Therefore, the Hint-W qPCR method can be easily scaled up to meet the needs of any laboratory, while other PCR-based methods may be more difficult to scale up for large-scale experiments. Additionally, our reactions were performed with low volume of SYBR Green (BioRad) reaction mix, leading to extra savings. The Hint-W qPCR method is the most cost-effective, based on current prices for commercially available products our method costs approximately 30$ per 100 samples (Table 1). Other methods are much more expensive except for the XhoI/18S (28) that is more labor intensive and less scalable. The crude blood lysate could also reduce the cost of other genotyping methods, for example, we used it for CHD-1 successfully. However, we did not test the other methods using crude lysate so they would have to be validated before large scale applications. These advantages make our method a more efficient, effective, and affordable option for chicken genotyping experiments.

**Table 1:** Comparison of the most relevant features of common PCR methods used for sex identification of chicken.

Primers	Target	Reference	Method	Specificity	Sensitivity	High-throughput applicability	DNA purification	Electrophoresis	Intensiveness of labor	Type of labor	Sample type	Estimated costs for 100 samples ($)
HTF1/HTR1	HINT-W	This study (28)	qPCR	+++	+++	+++	No	No	Low	Crude lysis	Crude lysate of blood	~30$
DMRT-1	DMRT-1	He et al. (17)	qPCR	+++	+++	+++	Yes	No	Low	gDNA purification	Purified DNA of brain tissue	~155$
P2/P8	CHD1-W/CHD1-Z	Griffiths et al. (15)	PCR	+	+	+	Yes	Yes	Moderate	gDNA purification/electrophoresis	Purified DNA	~190$
2250/2718	CHD1-W/CHD1-Z	Fridolfsson and Ellegren (26)	PCR	++	+++	+	Yes	Yes	Moderate	gDNA purification/electrophoresis	Purified DNA of blood or tissue	~190$
XhoI/18S	Chromosome W repeat sequence	Clinton et al. (19)	PCR	++	+++	+	No	Yes	Moderate	electrophoresis	Crude lysate of soft tissue	~36$
EE0.6/CPE or EE0.6/SINF	ET15	Itoh et al. (27)	PCR	+++	++	+	Yes	Yes	Moderate	gDNA purification/electrophoresis	Purified DNA of blood or tissue	~190$
SpinW TaqMan^®^	SPIN-W	Applied biosciences	qPCR	+++	+++	+++	Yes	No	Moderate	gDNA purification	Purified DNA of blood or tissue	390$

+, low; ++, good; +++, excellent; Table modified from He et al. (17).

## Conclusion

PCR is a powerful tool for genotyping organisms with high sensitivity and accuracy. However, one of the limitations of traditional chicken sexing methods is the DNA purification and low throughput processing, which results in longer experimental time and higher operational costs. Sample processing time is not a problem for research labs using small test groups, but for larger testing in the industry it is necessary to use a method with faster processing time. We designed one pair of primers that amplify one unique sequence from the female W chromosome. Then, we took advantage of avian nucleated red blood cells to create a quick lysate that has enough gDNA for a direct PCR reaction without need for DNA purification. Furthermore, we eliminated the gel electrophoresis that can process less samples at a time and instead implemented qPCR which can process 96 samples per run. Our method requires fewer steps than other chicken sexing protocols using PCR and has the lower cost per sample, which confirms it is ideal for processing large amount of samples. At last, we established the Ct-score obtained in the qPCR reaction that is required for precisely determining the sex of the chicken. In this report, we present a quick protocol suitable for large-scale and high-throughput genotyping of chicken sex.

## Data availability statement

The raw data supporting the conclusions of this article will be made available by the authors, without undue reservation.

## Ethics statement

The animal study was approved by Syracuse University Institutional Animal Care and Use Committee (IACUC). The study was conducted in accordance with the local legislation and institutional requirements.

## Author contributions

SF-M and CC contributed to conception and design of the study and wrote the first draft of the manuscript. VM, NG, AL, and AM preformed experiments and organized the database of different breeds. DV performed the statistical analysis. SD and JC provided critical scientific revision and wrote sections of the manuscript. All authors contributed to the article and approved the submitted version.
