# Supplementary material for: Fast, accurate, and cost-effective poultry sex genotyping using real-time polymerase chain reaction
Source: Front Vet Sci. 2023 Nov 3;10:1196755. doi: 10.3389/fvets.2023.1196755 (PMC10655105; doi:10.3389/fvets.2023.1196755)
Supplement: Supplementary file 1 [file Data_Sheet_1.DOCX]

Supplementary Material

Fast, accurate, and cost-effective poultry sex genotyping using Real-Time polymerase chain reaction

**Ciro D. Cordeiro, Nesim Gonceer, Steve Dorus, James E. Crill, Vardit Moshayoff, Amit Lachman, Asaf Moran, Dan Vilenchik, Shlomit Fedida-Metula***

*** Correspondence:** Corresponding Author: [shlomit@soos.org.il](about:blank)

## Supplementary Figures


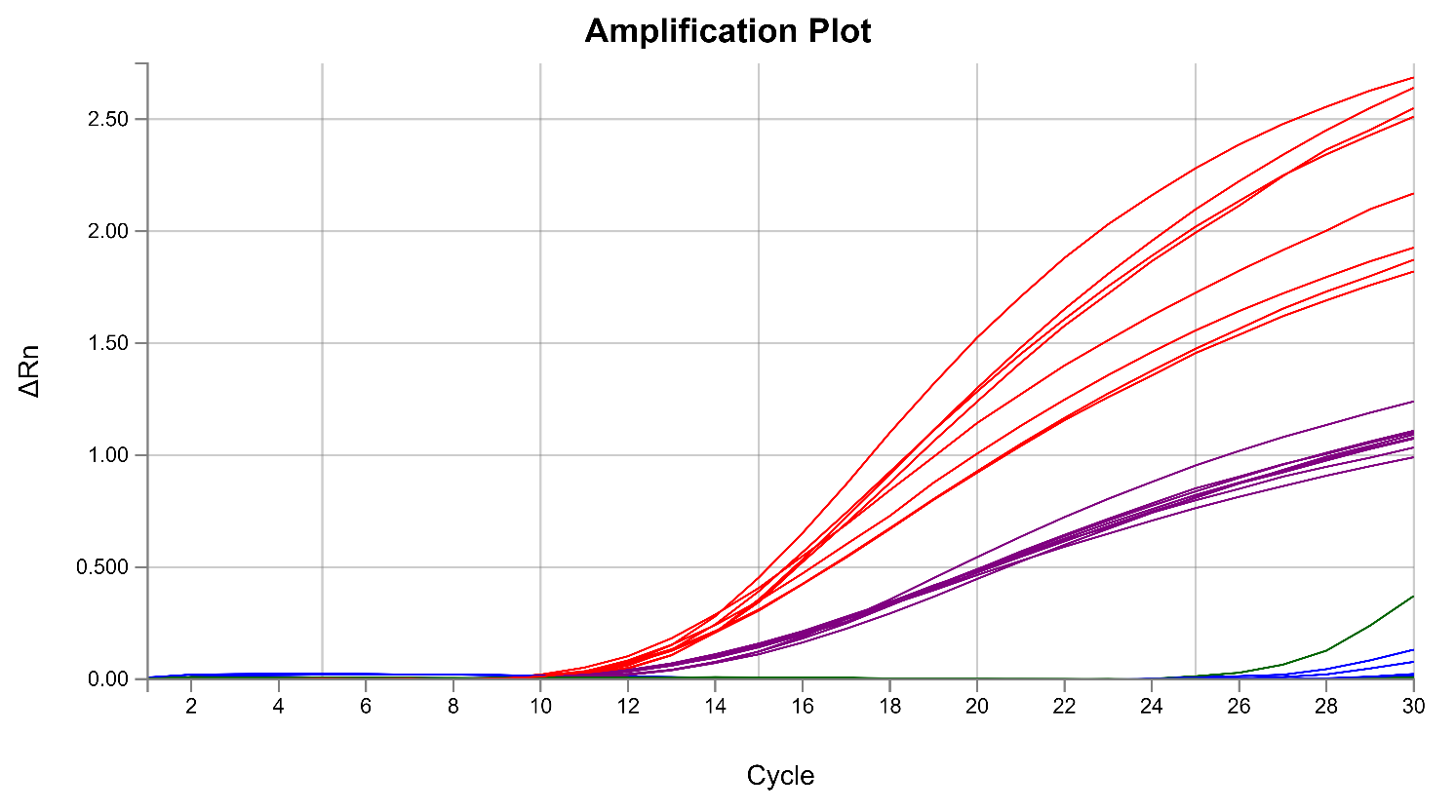


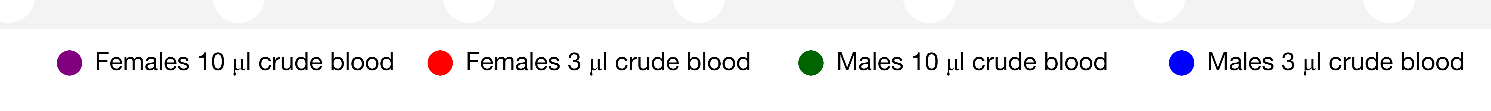

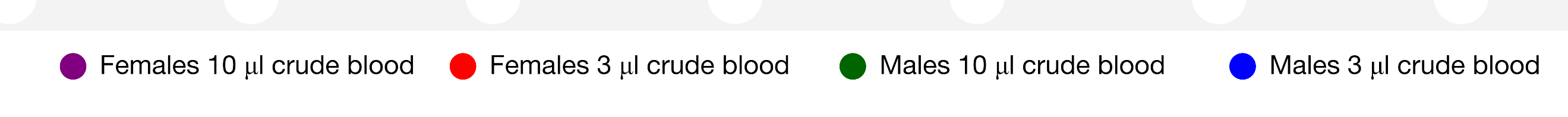


**Supplementary Figure 1.** qPCR amplification of crude lysates samples obtained from 3 µl or 10 µl of blood of males and females chicks.
