# Supplementary material for: Fast, accurate, and cost-effective poultry sex genotyping using real-time polymerase chain reaction
Source: Front Vet Sci. 2023 Nov 3;10:1196755. doi: 10.3389/fvets.2023.1196755 (PMC10655105; doi:10.3389/fvets.2023.1196755)
Supplement: Supplementary file 2 [file Data_Sheet_2.DOCX]

Supplementary Material

Fast, accurate, and cost-effective poultry sex genotyping using Real-Time polymerase chain reaction

**Ciro D. Cordeiro, Nesim Gonceer, Steve Dorus, James E. Crill, Vardit Moshayoff, Amit Lachman, Asaf Moran, Dan Vilenchik, Shlomit Fedida-Metula***

*** Correspondence:** Corresponding Author: [shlomit@soos.org.il](about:blank)

## Supplementary File 1: protocol for DNA sexing by qPCR.

**Blood sampling**

1. Using a 4 mm animal lancet, make a very small incision or a hole on the side of the leg finger and wait for a small drop of blood to start accumulate. Pay attention so that the blood drop is entirely blood with no lymph fluids (look for a good red blood drop with no transpernt liquids).
2. Using a pippett, draw ~3 µl of pure blood and place in a 96 well V-shape plate in the correct affiliated well.
3. Continue collecting blood until plate is full or until no samples are left.
4. Leave one well for internal negative control with no DNA but water instead.
5. Note: Put the sampled blood at the bottom of the tube and let it dry. Dried samples can be stored without cooling for long periods of time.

**Blood lysate**

1. Add 90 µl of 0.05M NaOH to each tube containing blood. Cover plate with adesive plastic cover.
2. Boil the samples (using PCR machine) to 95℃ for 10 minutes.
3. Let samples cool for a few minutes.
4. Shake gently until the samples are homogenus with no percipitants.
5. Spin down plate befor removing the plastic cover to avoid well-to-well contamination.
6. Add 10 µl of 1M TRIS (PH 7.5) to each well.
7. Shake gently for 20-30 seconds until samples are homogenus
8. Spindown the plates.
9. Take1 µl of each lysate for genotyping by qPCR.

If plate is kept in fridge, boil it again before use.

**qPCR analysis**

We use 10 µl total volume reaction using only 1 µl of total cell lysate for each reaction.

qPCR is performed accorrding to the manufacure recomendations (Bio-Rad):

prepare PCR master mix as follows:

|  | X1 | X100 (Full plate) |
| --- | --- | --- |
| iTaq universal SYBR Green Supermix (cat# 172-5122) | 5 µl | 500 µl |
| Water | 3.6 µl | 360 µl |
| Primer-Forward | 0.2 µl | 20 µl |
| Primer-Reverse | 0.2 µl | 20 µl |

1. Add 1 µl of lysate to each well.
2. Transfer 9 µl of qPCR mix to each well.
3. Seal the plate with plastic cover.
4. Spindown the plate.
5. Insert into the qPCR machine and start the following program:


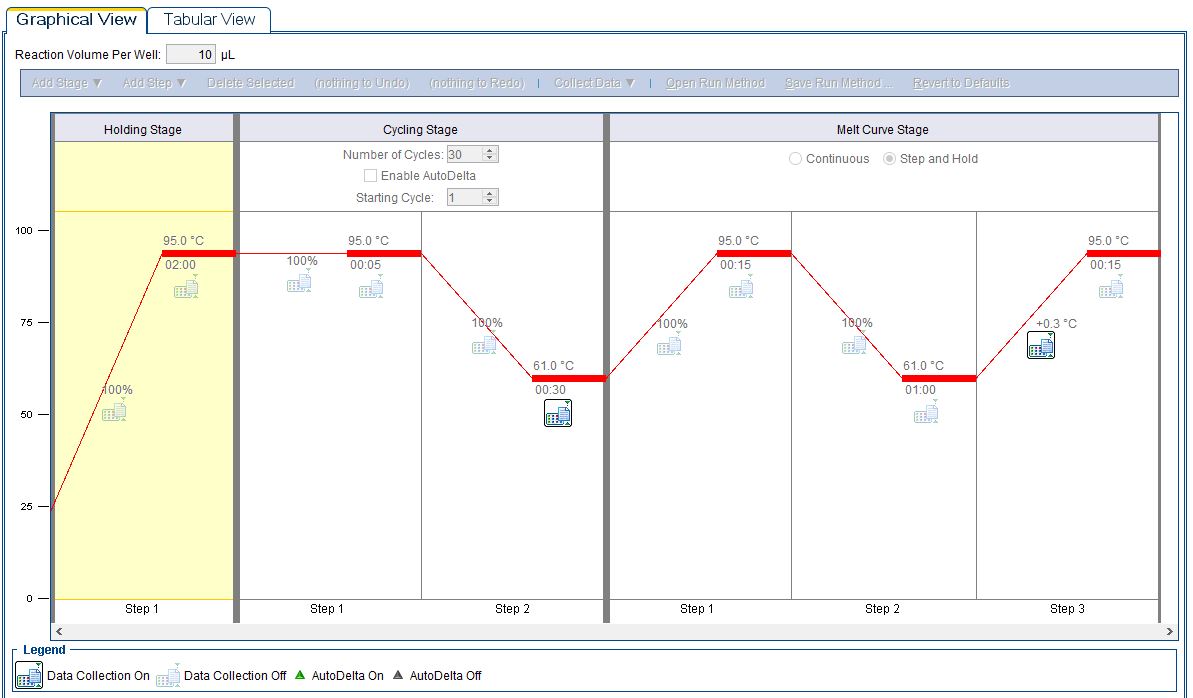


**Note: The above figure was taken from the StepOne Plus Real Time PCR machine by Applied Biosystems.**

1. Analyze the results and detrmine the DNA genotype of chicks.


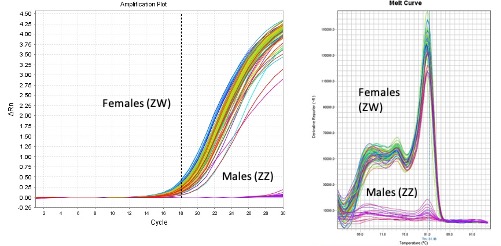


1. As a general rule, exponential slope in the female samples will apear after cycle #10-18 while in male samples, no amplification will be observed or in a much later cycles (above Ct 23) In any case, male samples amplification can be compared to blank control amplification since both should be negative.

**Refrences**

1. Hori, T. Asakawa, S. Itoh, Y. Shimizu, N. Mizuno, S. (2000) Wpkci, Encoding an Altered Form of PKCI, Is Conserved Widely on the Avian W Chromosome and Expressed in Early Female Embryos: Implication of Its Role in Female Sex Determination. Molecular Biology of the Cell Vol. 11, 3645–3660.
2. Smith, C.A. (2007). Sex Determination in Birds: HINT s from the W Sex Chromosome? Sex Dev 2007; 1:279–285.
